# Chemoradiation induces upregulation of immunogenic cell death-related molecules together with increased expression of PD-L1 and galectin-9 in gastric cancer

**DOI:** 10.1038/s41598-021-91603-7

**Published:** 2021-06-10

**Authors:** S. H. Petersen, L. F. Kua, S. Nakajima, W. P. Yong, K. Kono

**Affiliations:** 1 Cancer Science Institute of Singapore, National University of Singapore, Singapore, 117599 Singapore; 2 Department of Haematology-Oncology, National University Hospital of Singapore, Singapore, 119228 Singapore; 3 Department of Immunology, Faculty of Medicine, University of Yamanashi, Yamanashi, Japan; 4 Department of Gastrointestinal Tract Surgery, Faculty of Medicine, Fukushima Medical University, Fukushima, Japan; 5 Department of Progressive DOHaD Research, Faculty of Medicine, Fukushima Medical University, Fukushima, 1 Hikariga-oka, Fukushima city, Fukushima 960-1295 Japan

**Keywords:** Cancer, Immunology

## Abstract

Surgery alone or combined with chemo- and/or radiation therapy remains the primary treatment for gastric cancer (GC) to date and immunotherapeutic tools such as monoclonal antibodies are only slowly being implemented. This is partly due to the fact that the immune microenvironment in GC during chemoradiation and other treatment modalities is still poorly understood. 7 gastric cancer (GC) cell lines were tested for their response to chemoradiation using 5-FU in combination with X-ray irradiation. We conducted flow cytometric analysis to determine the cells’ ability to undergo immunogenic cell death (ICD) and their expression of the two immunosuppressive proteins programmed death-ligand 1 (PD-L1) and galectin-9 (Gal-9). We evaluated the overall immunogenicity of two cell lines (MKN7, MKN74) in co-culture experiments with human monocyte-derived dendritic cells (Mo-DCs). Chemoradiation induces distinct responses in different GC cell lines. We observe ICD in vitro in all tested GC cell lines in the form of calreticulin (CRT) translocation to the plasma membrane. As a resistance mechanism, these cells also upregulated Gal-9 and PD-L1. Mo-DC maturation experiments showed that GCs provoked the maturation of Mo-DCs after chemoradiation in vitro. The addition of α-PD-L1 blocking antibody further enhanced the immunogenicity of these cells while improving DC viability. Blocking Tim-3, as the main receptor for Gal-9, had no such effect. Our findings suggest that the benefits of chemoradiation can substantially depend on tumor subtype and these benefits can be offset by induced immune evasion in GC. Combination treatment using checkpoint inhibitors could potentially lead to enhanced immune responses and yield better patient outcomes.

## Introduction

GC represents the fifth most common cancer with the third most cancer-related deaths worldwide as of IARC-Globocan 2018. The worldwide occurrence of GC is highly variable with an incidence rate that is down markedly in western countries over the last few decades but remains notably prevalent in eastern Asia and eastern Europe. The current treatment of choice for GC remains curative resection in stage I–IVa GC and chemotherapy or chemoradiation therapy for more advanced stage tumors. Depending on tumor phenotype and condition of the patient, perioperative chemotherapy (before and after surgery), or preoperative chemoradiation is advised. Widely used regimens include combinations of 5-FU together with cisplatin or oxaliplatin (http://www.nccn.org). To find new treatment options, potential immunotherapeutic approaches are being investigated and are currently approved for monotherapy.

One of the best understood immune checkpoint pathways is PD-L1 on cancer cells binding to PD1 expressed on immune cells. This can lead to inhibition and apoptosis of T cells^1–9^. Ligation of PD-1 expressed on DCs, however, can also affect the latter’s function. This has been shown to lead to decreased expression of maturation markers and increased interleukin (IL)-10 production in DCs, suggesting the acquisition of a suppressive DC phenotype^10,11^ or the downregulation of their immune functionality^12,13^.

The increasing interest in immunotherapy also led to an increased understanding of the PD1–PD-L1 interface and its impact on the composition of the tumor microenvironment and immune evasion in GC^14–22^. Of interest for the current study, a direct correlation between GC treatment using 5-FU and the upregulation of exosomal PD-L1 was found recently^23^. Following clinical evidence of the beneficial effect of using PD-1 blocking antibody treatment^24–26^, pembrolizumab and nivolumab received clinical approval for GC treatment. Another checkpoint pathway is Gal-9 and its receptor T cell immunoglobulin and mucin-domain containing 3 (Tim-3), which is considered a co-inhibitory receptor on immune cells. Tim-3 has been shown to be overexpressed on exhausted T cells in chronic viral infections or cancer^27–31^ but is also present on DCs^32,33^ and has been shown to exert suppressive functions in that context as well^34^. Correspondingly, the secretion of soluble or exosome-bound Gal-9 by cancer cells, and the subsequent reattachment to cancer cell’s surface, supports cancer immune evasion and tumor progression in different malignancies^35–41^. Both Tim3 and PD1 receptor signaling are potential mechanisms of resistance against the potentiation of anticancer therapies by the immune system, such as through immunogenic cell death (ICD).

Generally recognized to be induced by endoplasmic reticulum (ER) stress, ICD is a functionally specialized form of apoptosis resulting in the regulated activation of the immune system by secretion or passive release of a variety of danger associated molecular patterns (DAMPs). The translocation of the endoplasmic protein CRT to the plasma membrane functions as an “eat-me-signal” for DCs and was found to be a main player in building immunogenicity^42–44^. Among 24 tested cytotoxic therapeutics, all of them equally caused apoptosis whereas only three anthracyclines and oxaliplatin were able to induce ICD. Apart from CRT, two more molecular characteristics have been identified as hallmarks of ICD, the secretion of ATP and the release of the cell death associated protein high-mobility group box 1 (HMGB1)^45–49^. The detection of these properties has proven to be sufficient to accurately predict the capacity of cytotoxic agents to induce ICD in cancerous cells. Such agents include cytostatics such as anthracyclines or oxaliplatin and other ER stress inducing treatments such as radiotherapy. ICD is known to stimulate an immune response against dead cell antigens, which is characterized by an initial dendritic cell (DC) stimulation followed by a cellular cascade that results in the activation of the innate as well as the adaptive immune response. In the context of cancer therapy, cross presentation of tumor-derived antigens via MHC class I molecules on DCs leading to an anticancer CD8^+^ T cell response is particularly relevant^50,51^. In line with this, treatment-driven ICD has been shown to give rise to anticancer immune responses strengthening the therapeutic effect of standard ICD-inducing chemo- and radiotherapies. Additionally, the combination with immunotherapies such as immune checkpoint inhibitors is gaining attention^52,53^. Clinically, however, only few ICD inducing treatments have been effectively utilized so far and more research has to be undertaken to further enable us to exploit these mechanisms to the patient’s benefit. Likewise, for GC, the current understanding of ICD induction and the potential utilization of such in clinical treatment regimens is poorly understood. There is little specific evidence of treatment induced ICD in GC except two clinical case reports describing ICD following radiation treatment^54,55^ and a study demonstrating honokiol to be an ICD inducer in GC^56^.

In the present study, we evaluated to what extent chemoradiation induced ICD in GC cell lines, whether we saw a difference in PD-L1 and Gal-9 expression, and how these mechanisms affected DC function. Here we show for the first time that chemoradiation using X-ray radiation combined with a single dose of 5-FU caused ICD in GC cell lines, as evidenced by the translocation of CRT. Additionally, we saw a significant upregulation of PD-L1 and Gal-9 with direct effects on DC maturation which could be attenuated using receptor specific blocking antibodies.

## Results

### Chemoradiation induces translocation of CRT and upregulation of PD-L1 and Gal-9 in HCT-116 and GC cells

**Figure 1:**
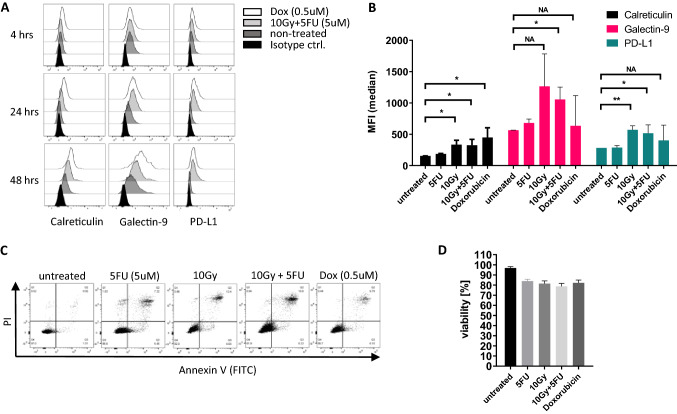
Chemo-/radiation treatment induced ICD in HCT116 cells. HCT-116 cells were treated with a single dose of 5FU (5 μM), X-ray irradiation (10 Gy), chemoradiation or DOXO (0.5 μM) and analyzed using flow cytometry. (**A**) Protein surface expression 4, 24 and 48 h after treatment, (**B**) bar graph of median fluorescence intensity 48 h after treatment, (**C**) apoptosis and (**D**) bar graph of viability 48 h after treatment. Bar graphs display the mean and SEM of at least 3 biological replicates. *P < 0.05, **P < 0.01, ***P < 0.001, *NA* not applicable (not significant).

Since ICD on HCT-116 cells (colon cancer) was reported to be induced with doxorubicin (DOXO) treatment^57–59^, doses of 5FU and irradiation intensity in the present study were chosen after conducting preliminary screening experiments with HCT-116. HCT-116 cells were treated with 5 µM 5-FU, a dose of 10 Gy X-ray irradiation, 5 µM 5-FU plus 10 Gy or 0.5 µM DOXO for 48 h. Surface expression levels of Calreticulin (CRT), Gal-9 and PD-L1 were measured 4, 24 and 48 h after treatment. We detected the highest level of CRT after 48 h, which we deemed to be the optimal length of time to measure induction of ICD (Fig. 1A). By comparing median fluorescent intensities (MFI), we detected a significant increase of CRT surface expression after combination treatment of 5-FU plus radiation (P = 0.035), radiation treatment alone (P = 0.011) and doxorubicin alone (P = 0.031). CRT surface upregulation was negligible after treatment with 5-FU alone (Fig. 1B). We also detected an increase in surface expression of Gal-9 and PD-L1 in HCT-116 cells treated with chemoradiation (P = 0.013 or P = 0.038, respectively). The average level of apoptosis after treatment was 21.3% for chemoradiation and 17.87% for DOXO (Fig. 1C,D).

**Figure 2:**
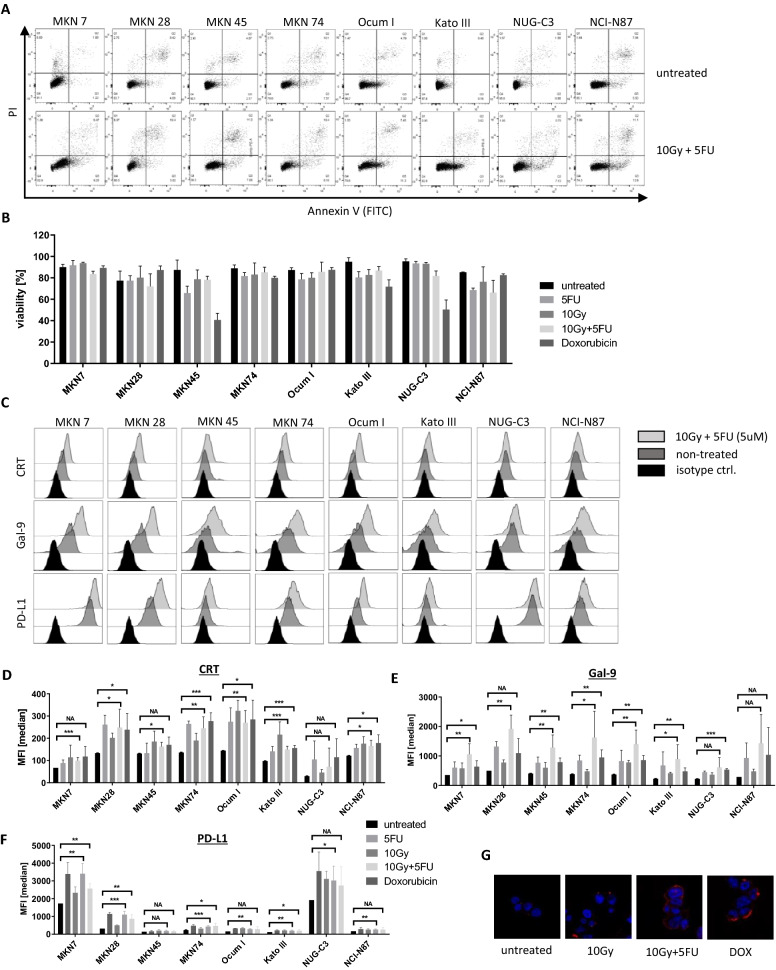
Chemo-/radiation induced ICD and upregulation of Gal-9 and PD-L1 in GC cell lines. Eight GC cell lines were treated with a single dose of 5FU (5 μM), X-ray irradiation (10 Gy), chemoradiation or DOXO (0.5 μM) and analyzed 48 h after single dose treatment. (**A**) Flow cytometry data displaying level of apoptosis in each cell line for untreated and chemoradiation treated cells, (**B**) bar graph of viability of GC cell lines for each condition, (**C**) representative flow cytometry data for protein expression of CRT, Gal-9 and PD-L1 in each cell line, either untreated or after chemoradiation, (**D**–**F**) bar graphs displaying MFI for CRT, Gal-9 and PD-L1, respectively, (**G**) immunofluorescent imaging of CRT expression in MKN74 cells. Bar graphs display the mean and SEM of at least 3 biological replicates. *P < 0.05, **P < 0.01, ***P < 0.001, *NA* not applicable.

The same conditions were tested on the GC cell lines, MKN7, MKN28, MKN45, MKN74, Ocum I, Kato III, NUG-C3 and NCI-N87. Most of these cell lines were highly resistant to the chosen dose of 5-FU, 10 Gy of radiation or combination treatment as seen in low apoptosis rates (Fig. 2A,B). However, we observed a significant translocation of CRT to the surface after chemoradiation in all tested cell lines except NUG-C3 (Fig. 2C,D,G). Simultaneously, Gal-9 was significantly upregulated for all cell lines except NUG-C3 and NCI-N87 (Fig. 2E). Similar results were obtained using Oxaliplatin (data not shown).

Surface expression of PD-L1 was highly heterogeneous among the tested GC cell lines. MKN7 and NUG-C3 showed the highest natural PD-L1 expression by far before and after treatment (Fig. 2F). We noted that these two cell lines also had the lowest baseline CRT expression (Fig. 2D).

Taken together, chemoradiation generally induced translocation of CRT together with upregulation of PD-L1 and Gal-9 in GC cells, although the expression levels were heterogenous.

### Chemoradiation causes ER-stress-induced pre-apoptotic CRT translocation in MKN7 and MKN74 cells

We chose to continue to focus this study on the cell lines MKN7 and MKN74 due to their significant CRT translocation upon treatment and their markedly different profile in PD-L1 expression. First, we established the sensitivity of these two cell lines to 5-FU and compared this to HCT-116 cells. 48 h after the addition of 5-FU, MTT assays were conducted, which revealed a high resistance to 5-FU in MKN74 and MKN7 cells. Whereas the half maximal inhibitory concentration (IC50) of 5-FU in HCT-116 cells was ~ 19 μM, the IC50 was ~ 110 µM for MKN74 and a above 2000 µM for MKN7 (Sup. Fig. 1A–C).

Using higher concentrations of 5FU, viability was not much affected in either MKN7 (Sup. Fig. 2A,B) or MKN74 cells (Sup. Fig. 2D,E). However, the combined treatment (5FU + irradiation) led to a synergistic effect in causing a G2 cell cycle arrest. MKN7 cells in G2 increased from 28% in untreated cells to 54.97% (P = 0.0022) in cells treated with 150 µM 5-FU plus 20 Gy and to 51.2% (P = 0.0056) in cells treated with 300 μM plus 20 Gy of radiation (Sup. Fig. 2A,C). In MKN74, we observed an increase of cells in G2 phase from 8.15 to 21.6% (P = 0.0058) for the treatment combination of 75 µM 5-FU plus 20 Gy, and to 26.17% (P = 0.007) for 150 µM 5-FU plus 20 Gy (Sup. Fig. 2D,F).

**Figure 3:**
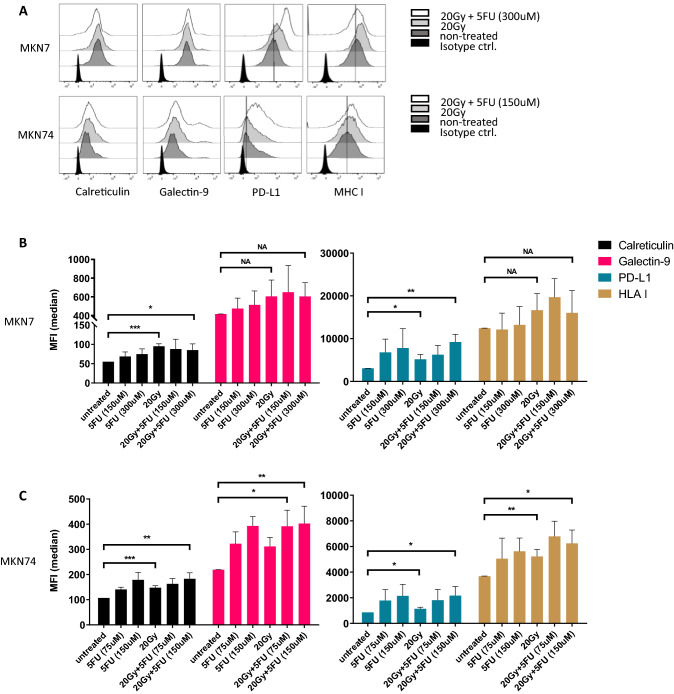
MKN7 and MKN74 elicit cell cycle arrest and upregulation of CRT, Gal-9 and PD-L1. MKN7 and MKN74 cells were treated with 75 μM, 150 μM or 300 μM 5FU, 20 Gy of X-ray irradiation or combinations of these. 48 h after treatment they were stained using PI and AnnexinV to analyze apoptosis or PI in permeabilized cells to analyze cell cycle phase. Surface proteins were stained and analyzed by flow cytometry as above. (**A**) Surface protein expression in MKN7 and MKN74 after radiation or chemoradiation treatment. (**B**) Bar graph of MFI of surface protein expression in MKN7 and (**C**) MKN74 cells. Bar graphs display the mean and SEM of at least 3 biological replicates. *P < 0.05, **P < 0.01, ***P < 0.001, *NA* not applicable.

We observed a significant translocation of CRT after combination treatment compared to untreated cells (MKN7: P = 0.031, MKN74: P = 0.0053). PD-L1 expression was significantly increased in both cell lines (MKN7: P = 0.0042, MKN74: P = 0.036, Fig. 3A–C). Surface expression of Gal-9 and HLA class I were also upregulated in a similar fashion (Fig. 3A–C). We did not detect the presence of soluble Gal-9 or HMGB1 in conditioned media by ELISA (data not shown). The mRNA expression of Gal-9 and PD-L1 was not upregulated despite a higher surface expression. We only saw an increase in CRT mRNA, which was expected during ER-stress (Sup. Fig. 3B). Similarly, we did not see an increase in total protein expression for CRT and PD-L1 using WB analysis (Sup. Fig. 3C). The RNA expression of proteins involved in ER-stress response in MKN74 cells verified the induction of ER stress after an incubation time of 48 h (Sup. Fig. 3A).

### Chemoradiation of MKN7 and MKN74 and PD-L1 blocking synergistically induce Mo-DC maturation

**Figure 4:**
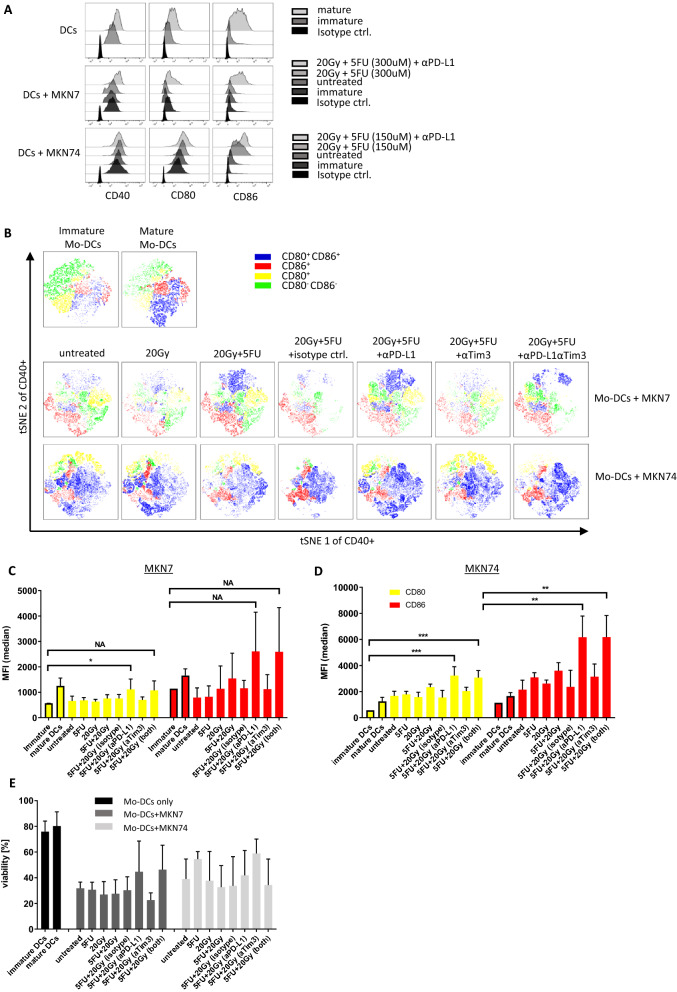
MKN7 and MKN74 induce maturation in DCs after chemo-/radiation treatment. MKN7 and MKN74 were treated using 150 μM or 300 μM of 5FU, 20 Gy of X-ray irradiation or chemoradiation. After 48 h of culture they were combined with immature Mo-DCs isolated from PBMCs. (**A**) Surface protein staining of Mo-DCs after 72 h of co-culture, (**B**) tSNE analysis of concatenated replicates of Mo-DCs co-cultured with either MKN7 or MKN74 cells, (**C**,**D**) bar graphs of MFI of surface proteins staining in Mo-DC co-cultured with either MKN7 or MKN74, respectively, (**E**) bar graph of viability of Mo-CDs. Bar graphs display the mean and SEM of at least 3 biological replicates. *P < 0.05, **P < 0.01, ***P < 0.001, *NA* not applicable.

We next treated MKN7 and MKN74 cells with either 300 µM or 150 µM of 5-FU plus a single dose of 20 Gy of radiation. After 48 h, immature Mo-DCs were mixed with the treated GC cells. These cells were co-cultured for an additional 72 h after which we measured the surface expression of the maturation markers CD40, CD80 and CD86 on DCs by flow cytometry. All three markers were upregulated upon maturation using commercial maturation medium as a positive control condition (Fig. 4A,B). The same trend can be seen for immature Mo-DC co-cultured with treated GCs (Fig. 4A,B). Dimensionality reduction analysis using t-distributed stochastic neighbor embedding (t-SNE) on concatenated biological replicates helped us visualize the overall level of maturation in Mo-DCs (Fig. 4B). Gating for CD80 and CD86 expressions, we see a clear shift from predominantly CD80^−^ CD86^−^ cells in immature Mo-DCs towards a strong CD80 and/or CD86 expression in mature Mo-DCs. Co-culture with MKN7 cells, treated or untreated, had no significant effect on Mo-DC. Adding α-PD-L1 blocking Abs, however, enhanced the maturation of DCs as seen by a marked increase in CD80^+^ CD86^+^ cells. Interestingly, this effect was less pronounced when we added α-Tim3 blocking Abs or a combination of both (Fig. 4B). MKN74 cells, on the other hand, strongly stimulated Mo-DCs, even when untreated, leading to a mature phenotype in a large majority of DCs. Radiation or chemoradiation of MKN74 cells prior to co-culture had an additional stimulatory effect seen by a further increase of double-positive DCs. Due to this strong stimulatory effect of MKN74 cells, our chosen tSNE analysis was unable to decipher any marked additional benefit of adding blocking Abs to these co-cultures (Fig. 4B).

As expected, we saw a significant MFI increase in CD80 and CD86 expression for mature Mo-DCs compared to their immature counterpart (P = 0.0044 and P = 0.0084, respectively). MKN7 cells again proved to have no stimulatory effect whether untreated or 5-FU treated. We saw a marginal increase in both markers using MKN7 cells treated with radiation or chemoradiation. A strong stimulatory effect could, however, be observed after the addition of 10 µg/ml blocking α-PD-L1 Ab to the co-culture (Fig. 4C). This led to a significant upregulation of CD80 (P = 0.04) as well as a marked upregulation of CD86 (Fig. 4C). MKN74, as was seen above, demonstrated an inherent ability to stimulate DCs. This effect was slightly enhanced upon chemoradiation and significantly enhanced after the addition of α-PD-L1 blocking Ab (CD80: P = 0.0005, CD86: P = 0.0014) or in combination with α-Tim3 (CD80: P = 0.0002, CD86: P = 0.0015) (Fig. 4D). As seen in MKN7 cells, blocking the Gal-9-Tim-3 pathway alone did not show any beneficial effect on DC stimulation.

Co-culture of MKN7 or MKN74 with Mo-DCs caused substantial stress on the latter as seen in strongly decreased viability (Fig. 4E). Blocking PD-L1 on MKN7 cells apparently improved the viability of DCs, although the effect was statistically insignificant. Compared to an average of 27.5% of viable DCs after co-culture with chemoradiated MKN7 cells, the addition of α-PD-L1 or in combination with α-Tim3 increased this value to a mean of 44.6% and 46.3%, respectively. In the co-culture with MKN74 cells, blocking these checkpoints did not have this effect. Cell cycle phase of Mo-DCs was not affected by the co-culture of GC cell lines of any of these treatments (data not shown).

These results strongly indicate that chemoradiation induced cell stress in GC cell lines was capable of inducing ICD which in turn leads to DC maturation in co-culture. Furthermore, PD-L1 is a strong limiting factor in GC-dependent DC stimulation and blocking this ligand can significantly improve the DC maturation induced by treated GCs.

## Discussion

ICD has been researched extensively over the last decade providing us with a detailed molecular understanding of the mechanisms and pathways involved. It has been proven to occur in different cancer cells in vitro as well as in vivo and it is thought to play a crucial role in breaking tumor immune evasion. For GC specifically, however, we still know very little about the ability of frequently used treatments to induce ICD and what impact that has on current GC therapies. Here, we show for the first time that one of the most commonly used treatments, chemoradiation, induced ICD in GC cell lines. Despite the fact that the 7 tested GC cell lines did not display a high level of apoptosis 48 h post-treatment, we detected ICD-induced ER-stress and translocation of CRT to the cell surface. 5-FU and radiation each were able to induce ICD. Moreover, combined chemoradiation proved to be as efficacious in inducing ICD in GC cells as doxorubicin, one of the most potent ICD inducers currently known. In this study we decided to focus on high doses of chemoradiation as these resulted in a clearer distinction between untreated and treated samples. This does not exclude the possibility that lower doses as seen in Fig. 2 would result in similar effects on the cancer immune microenvironment to the ones we propose using high doses.

As a potential resistance mechanism to treatment, we detected the upregulation of PD-L1 and Gal-9 on the plasma membrane after chemoradiation, pointing to a simultaneous enhancement of immune evasion. Whereas, the expression and upregulation of Gal-9 was rather similar across all cell lines, the basic expression and subsequent upregulation of PD-L1 varied remarkably between the tested GC cell lines. Of particular note, MKN7 and NUG-C3 stood out with their vastly increased PD-L1 expression while displaying the lowest CRT surface expression among all tested cell lines. These two cell lines may represent GC phenotypes of very low immunogenicity in patients. Potential differences in the expression of ER-stress response genes or genes regulating JAK/STAT signaling could give mechanistic clues and would be interesting to be investigated in the future.

We further focused our research on comparing MKN7 as a potentially immune evasive cell line to MKN74 as a potentially immunogenic cell line. Examining the cells’ response to chemoradiation in more detail, we observed G2 cell cycle arrest (Sup. Fig. 2A–F) which, combined with CRT translocation, strongly indicates a pre-apoptotic state. This again was accompanied with Gal-9 and PD-L1 upregulation. Interestingly, HLA class I surface expression was equally elevated post-treatment, adding another level of complexity to the cancer cell’s immunogenicity. HLA class I upregulation on cancer cells after radiation has been seen before and, additionally to ICD, is likely to play a highly beneficial role in the induction of immunogenicity since it facilitates antigen specific CD8^+^ T cell dependent tumor cell killing^60–62^.

While CRT translocation is a well-established result of the unfolded protein response (UPR), a change in Gal-9, PD-L1 or HLA I expression may also be explained as a consequence of ER stress. Cellular stress-dependent changes in expression of Gal-1 transcripts have been shown earlier^63^; a similar pathway may also be responsible for the Gal-9 upregulation we observed in GC cells. The link between ER stress and PD-L1 as well as HLA I expression is more well-established. On the one hand, in contrast to our findings, previous data demonstrate that ER-stress is commonly associated with a suppression of MHC-I expression as well as MHC-I-peptide loading^64,65^. On the other hand, the IFNγ-driven JAK/STAT1 pathway is known to not only upregulate HLA I^66,67^ but also positively regulate the expression of PD-L1 in different cancer types including GC^68–70^. This is potentially downstream of UPR activated PERK signaling and capable to activate the JAK1/STAT3 pathway in murine astrocytes^71^, which makes a link between ER-stress and PD-L1 and HLA I upregulation probable. In support of this hypothesis is the fact that another UPR-dependent pathway, the IRE1/XBP1 pathway, is known to cause upregulation of PD-L1 in KSHV infected cells^70^.

A different explanation could be the evidence, although not fully elucidated yet, that the UPR can lead to enhanced protein export, depending on the intensity of the stress stimulus. This mechanism is thought to be a strategy by which UPR alleviates ER stress^72^ and would be more in line with our observation of a steady or reduced PD-L1 and Gal-9 production in MKN74 cells.

Corresponding to their different phenotype, particularly in surface expression of CRT and PD-L1, MKN7 and MKN74 showed distinct capacities to stimulate immature Mo-DCs upon co-culture. Whilst MKN7 cells on their own provoked a marginal maturation in DCs, the treatment with radiation or chemoradiation did not improve their capacity to cause DC maturation despite a significantly increased display of CRT compared to its basal level. MKN74, naturally higher in their CRT surface expression but dramatically lower in their PD-L1 expression, were able to provide a strong DC maturation stimulus in their untreated condition. Chemoradiation and subsequent CRT translocation did not lead to a significantly higher level of maturation indicating that concomitant increase in PD-L1 and/or Gal-9 may act as additional inhibitory signals.

Blocking PD-L1 proved this immune checkpoint to be a crucial player. Both cell lines provided a far better maturation stimulus to Mo-DCs in the presence of PD-L1 blocking Abs. For MKN7 cells, which have exceptionally high expression of PD-L1, blocking this ligand also positively improved DC viability. PD-L1 upregulation therefore may be a GC mechanism of resistance to chemoradiation induced ICD. Blocking the Gal-9-Tim3 interface, however, had no effect on either maturation or viability of DCs after co-culture indicating a far lesser impact on immunogenicity in respect to DC biology. We observe somewhat improved viability in DCs cultured with MKN74 in the presence of Tim-3 blocking Ab, but this was not significant and would have to be tested further. Additionally, PD1 and Tim-3 being crucial immune checkpoint receptors on T cells, future studies will have to include T cell killing assays as well as complex immunological assays which are going beyond DC stimulation.

Overall, we demonstrate that the PD-L1 expression can differ strongly between GC cell lines depending on the exact tissue of origin and genetic background. We provide new evidence of PD1-PD-L1 interaction potentially being a highly important player in GC immune evasion. These data emphasize not only the value of pembrolizumab or nivolumab being an approved treatment option for GC but also motivate further research into potential combination therapies involving current chemo-, radiation, or chemoradiation therapy regimens and immunotherapy.

## Materials and methods

### Ethics approval

This study was approved by the Ethics Committee of the National University of Singapore. All participants provided written informed consent for providing their blood donation to this study, and the methods were carried out in accordance with the approved guidelines.

### Cell lines and antibodies

All GC cell lines used in this study were acquired from the Japanese Collection of Research Bioresources Cell Bank (JCRB Cell Bank), Japan. HCT-116 cells were acquired from American Type Culture Collection (ATTC), US. GC cell lines were cultured in RPMI 1640 media (Gibco, 11875093) supplemented with 10% fetal calf serum (FCS, Gibco, 16010159) and 1% Penicillin/Streptomycin (Gico, 15140122). HCT-116 cells were cultured in DMEM media (Gibco, 11965092) supplemented as seen above.

Western blotting: αPD-L1 (purified; abcam, ab213524), αCalreticulin (purified; abcam, ab227444), αActin (purified; abcam, ab179467), αMouse (HRP; Thermofisher, G-21040), αRabbit (HRP; Thermofisher, G-21234).

Immuno-fluorescence: αCalreticulin (purified; abcam, ab227444), αRabbit (AF647; polyclonal, Invitrogen, A27040).

Flow cytometry: αCD14 Ab (FITC; Miltenyi Biotec, Mo-CD Differentiation Inspector, 130-093-567), αCD83 (APC; Miltenyi Biotec, Mo-CD Differentiation Inspector, 130-093-567), αCD209 (PE; Miltenyi Biotec, Mo-CD Differentiation Inspector, 130-093-567), αCD80 (APC; Miltenyi Biotec, 130-122-928), αCD86 (FITC; Miltenyi Biotec, 130-108-044), αCD40 (PE; Miltenyi Biotec, 130-123-952), αHLA class I (W6/32, PerCP-Cy5.5; Biolegend, 311420), αCalreticulin (PE; abcam, ab209577), αGalectin-9 (PerCP-Cy5.5; Biolegend, 348909), CD274 (αPD-L1, FITC; Biolegend, 393605).

### Generation of monocyte-derived dendritic cells (Mo-DCs)

Whole blood samples were acquired from healthy donors (Singapore General Hospital). The use of blood from healthy donors was approved by the Domain-Specific Review Board of the National University Hospital of Singapore (DSRB no. 2015/00031). Peripheral Blood Mononuclear Cells (PBMCs) were isolated using Ficoll-Paque (GE Healthcare, Little Chalfont, U.K.). Human monocytes (hPBMs) were isolated using MACS magnetic cell separation systems (Miltenyi Biotec) and CD14 MicroBeads (Miltenyi Biotec, 130050201).

Purified monocytes were cultured for 7 days in Mo-DC Differentiation Medium (Miltenyi Biotec, 130094812) to generate immature Mo-DCs. Immature Mo-DCs were cultured in Mo-DC Maturation Medium (Miltenyi Biotec, 130094813) for 3 consecutive days to generate mature Mo-DCs.

### Chemo-/radiation treatment

Cell lines grown in culture flasks or plates were irradiated using the X-ray Biological Research Irradiator “RS 2000” (Rad Source Technologies). The growth medium was then replaced with new medium containing the respective concentration of cytotoxic agent and was cultured for an additional 48 h unless stated otherwise. 5-Fluorouracil, Doxorubicin and Oxaliplatin were provided by the National University Hospital Singapore.

### Western blotting

Cell pellets were resuspended and lysed for 30 min on ice in RIPA lysis buffer supplemented with Protease/Phosphatase Inhibitor (1:100, Thermo Fisher Scientific, Massachusetts, USA). Protein concentration was determined using the BCA Protein Assay Kit (Pierce—Thermo Fisher Scientific, Massachusetts, USA) when necessary. Lysate was denatured by adding 4 × Laemmli Buffer (Biorad, California, USA) supplemented with 10% β-mercapto-ethanol and subsequent heat treatment at 95 °C for 10 min. Proteins were separated by SDS-PAGE. Samples were loaded onto precast gels (Invitrogen) and transferred to polyvinylidene difluoride membranes. The membranes were incubated in PBS-T (5% milk) blocking solution for 2 h at room temperature, followed by incubation with the respective monoclonal Ab, overnight in the dark at 4 °C. Each membrane was then washed three times with PBS-T and incubated with HRP-conjugated anti-rabbit Ab for 30 min at room temperature. Immunoreactive proteins were visualized using ECL Prime and/or ECL Select (GE Healthcare).

### Immunofluorescence

After treatment, cells were fixed with 0.25% ice cold paraformaldehyde (Electron Microscopy Sciences, Pennsylvania, USA) and permeabilized with PBST (PBS with 0.1% TritonX-100, Bio-Rad, California, USA). They were then blocked with 3% goat serum (Sigma-Aldrich, Merck, Darmstadt, Germany) in PBST and incubated with the primary antibody for 2 h. Finally, slides were mounted on glass slides using ProLong Gold antifade reagent (Invitrogen), and images were obtained using the Nikon A1R confocal microscope (Nikon, Tokyo, Japan).

### Flow cytometry

For immune-phenotyping, cells were stained with fluorescein-conjugated monoclonal antibodies. Viability was assessed using Life/Dead fixable stain (Thermo Fisher Scientific, Massachusetts, USA). Cells were incubated with Life/Dead stain in PBS (1:1000) for 10 min at 4 °C, then washed with PBS and incubated with the respective antibodies diluted in staining buffer (PBS, EDTA 2 mM, Hepes 15 mM, FCS 1:50) for 30 min at 4 °C in the dark. After incubation, cells were washed with staining buffer, resuspended in PBS, acquired using a BD LSR II flow cytometer (Becton Dickinson, BD, New Jersey, USA) and analyzed using the software Flowjo (Becton Dickinson, BD, New Jersey, USA).

### Apoptosis and cell cycle analysis

Apoptosis and cell cycle phase in cells were determined using the Multi Parameter Apoptosis Assay Kit (Cayman Chemical, 601280) and the Cell Cycle Phase Determination Kit (Cayman Chemical, 10009349) following the manufacturer’s manual instructions. Stained cells were acquired and analyzed as described above.

### Cell proliferation assay

The half maximal inhibitory concentration (IC50) of cytotoxic compounds was measured using the MTT Assay Kit (Cell Proliferation, abcam, ab211091). The kit was used on treated and untreated cells following the manufacturer’s manual instructions.

### Isolation of total RNA

Total RNA was isolated as previously described by Nakajima et al.^73^. Human peripheral blood monocytes, and cell lines were treated using TRIzol (Invitrogen) or a RNeasy Mini kit (Qiagen) according to the manufacturers’ instructions. RNA was quantified using a NanoDrop ND-1000 spectrophotometer (Thermo Fisher Scientific, Wilmington, DE), and the quality was verified by agarose gel electrophoresis.

### Quantitative PCR

cDNA was synthesized as previously described by Nakajima et al.^73^ using an iScript cDNA synthesis kit (Bio-Rad, 1708891). Semiquantitative PCR was performed on a Veriti 96-well thermal cycler (Applied Biosystems, Carlsbad, CA) using FastStart Taq DNA polymerase and dNTPack (Roche, Basel, Switzerland). Quantitative real-time PCR (qPCR) was performed on an ABI 7500 Fast real-time PCR system (Applied Biosystems) using SYBR Fast qPCR Master mix (Kapa Biosystems, Wilmington, MA). The quantitative PCR data were normalized relative to housekeeping genes, namely GAPDH. Sequences of primers can be provided upon request.

### Statistical analysis

Any data presented derive from at least 3 independent experiments and are expressed as means ± SEM. Multiple *t* tests were performed to determine statistical significance between two data sets, and differences were considered to be significant at P < 0.05.

## Supplementary Information

Supplementary Figures.
