# Supplementary material for: Chemoradiation induces upregulation of immunogenic cell death-related molecules together with increased expression of PD-L1 and galectin-9 in gastric cancer
Source: Sci Rep. 2021 Jun 10;11:12264. doi: 10.1038/s41598-021-91603-7 (PMC8192931; doi:10.1038/s41598-021-91603-7)
Supplement: Supplementary file 1 — Supplementary Figures. [file 41598_2021_91603_MOESM1_ESM.pdf]

Original Article:

Chemoradiation induces Upregulation of Immunogenic Cell Death-related Molecules together with increased Expression of PD-L1 and Galectin-9 in Gastric Cancer

Petersen S.H.[1], Kua L.F.[1], Nakajima S.[3,4,5,6], Yong W.P.[1,2], Kono K.[7*]

Cancer Science Institute of Singapore, National University of Singapore, Singapore 117599, Singapore

Department of Haematology-Oncology, National University Hospital of Singapore, Singapore 119228, Singapore

Department of Immunology, Faculty of Medicine, University of Yamanashi, Yamanashi, Japan

Department of Gastrointestinal Tract Surgery, Faculty of Medicine, Fukushima Medical University, Fukushima, Japan

Department of Progressive DOHaD Research, Faculty of Medicine, Fukushima Medical University, Fukushima, Japan

Department of Progressive DOHaD Research, Department of Gastrointestinal Tract Surgery, School of Medicine, Fukushima Medical University, 1 Hikariga-oka, Fukushima City, Fukushima, 960-1295, Japan

Department of Gastrointestinal Tract Surgery, Faculty of Medicine, Fukushima Medical University, Fukushima, Japan

Corresponding author: Kono K., Department of Gastrointestinal Tract Surgery, Faculty of Medicine, Fukushima Medical University, Fukushima, Japan, Email: kojikono@fmu.ac.jp TEL&FAX +81-24-547-1980

Sup. Fig. 1: MKN7 and MKN74 are strongly resistant to 5FU induced cell death

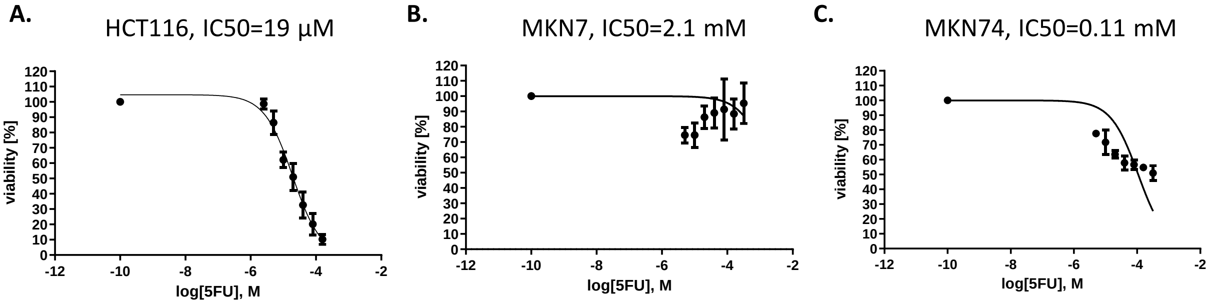

Sup. Fig. 1: Representative graphs of MTT assay to determine the IC50 of 5FU for A) HCT-116, B) MKN7 and C) MKN74. SEM of each data point represents 3 technical replicates.

Sup Fig. 2: MKN7 and MKN74 combine cell cycle arrest with upregulation of CRT, Gal-9 and PD-L1

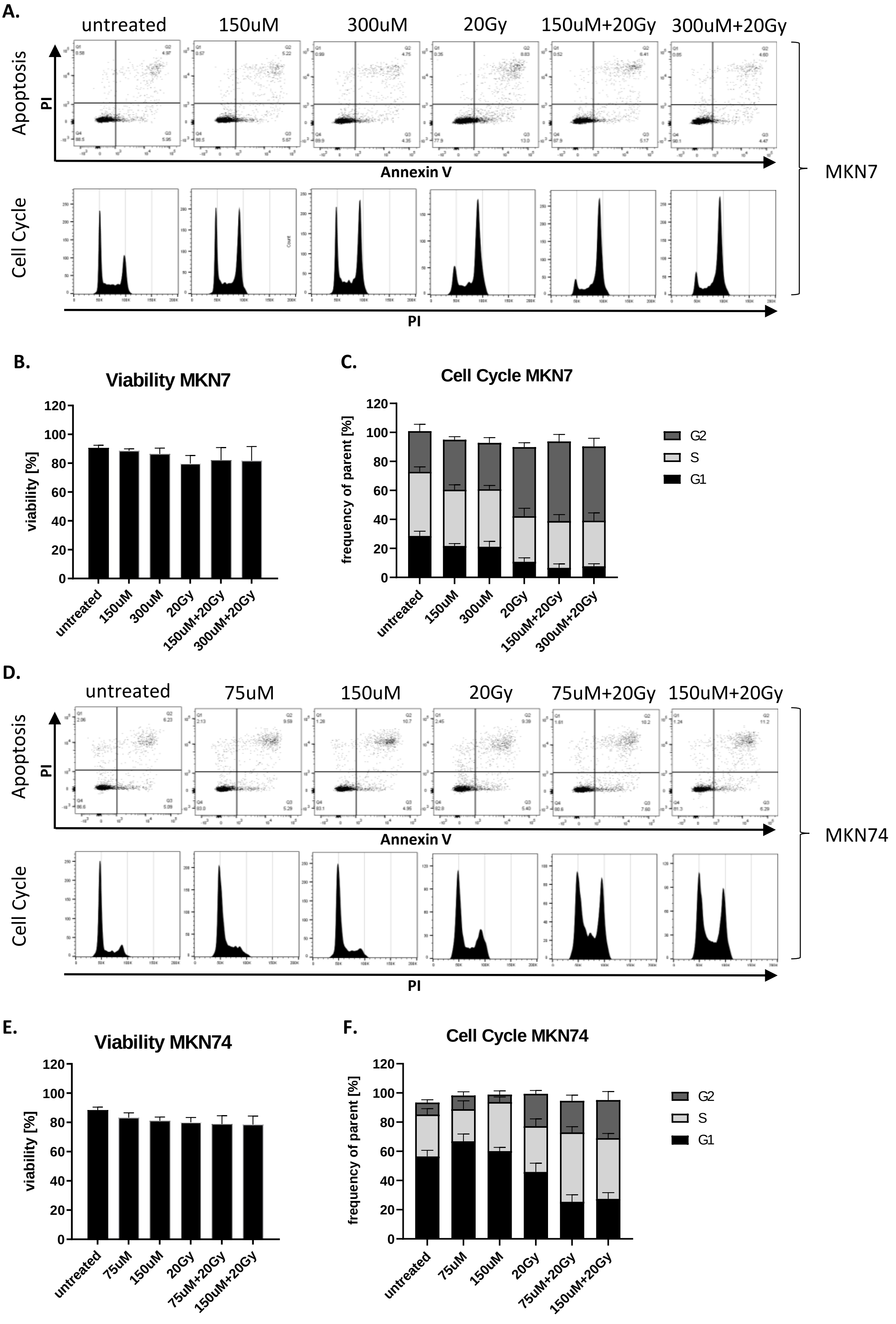

Sup. Fig. 2: MKN7 and MKN74 cells were treated with 75um, 150uM or 300uM 5FU, 20Gy of X-ray irradiation or combinations of these. 48 hrs after treatment they were stained using PI and Annexin V to analyse apoptosis or PI in permeabilised cells to analyse cell cycle phase. Surface proteins were stained and analyzed by flow cytometry as above. A-C) Apoptosis and cell cycle phase of MKN7 and D-F) MKN74 cells. Bar graphs display the mean and SEM of at least 3 technical replicates. PI: propidium iodide

Sup. Fig. 3: Chemoradiation treatment

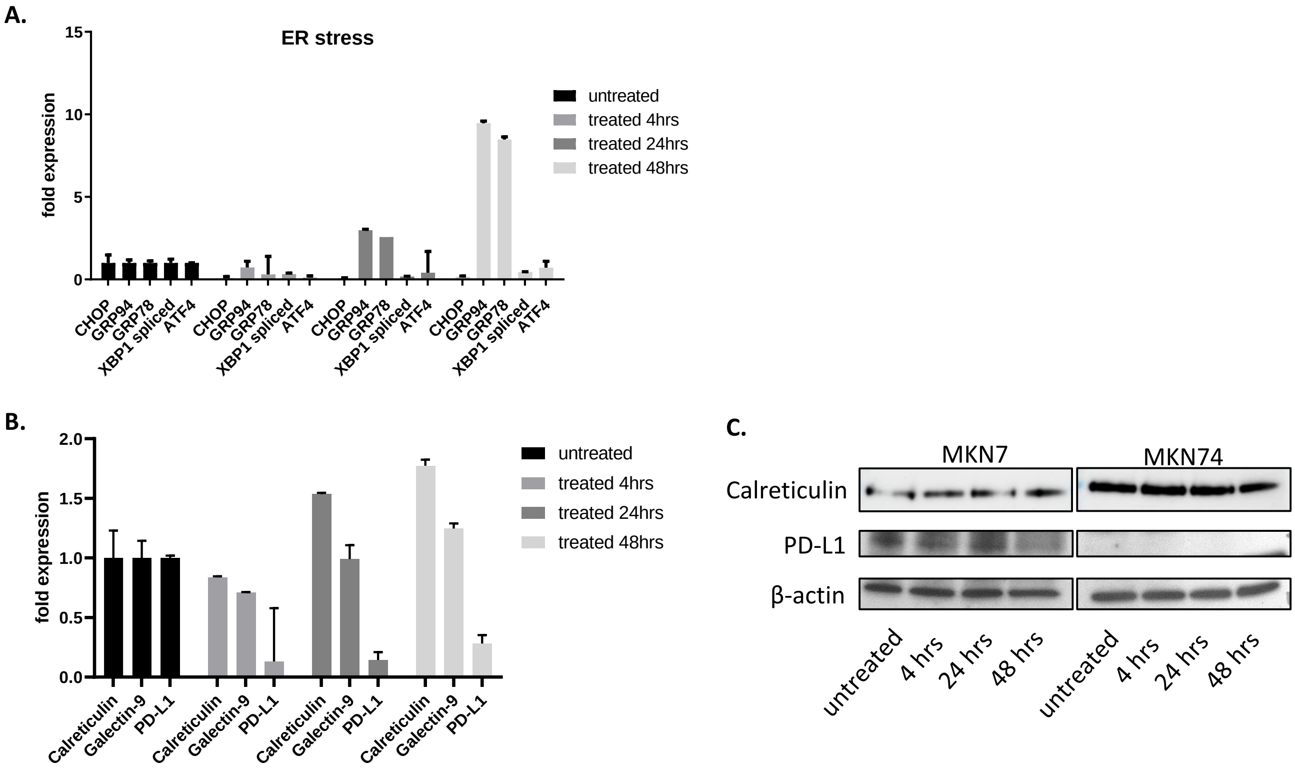

Sup. Fig.3: MKN7 and MKN74 were treated using 150uM or 300uM of 5FU, 20Gy of X-ray irradiation or chemoradiation and analysed 4, 24 and 48 hrs after single dose treatment. A) MKN74 mRNA expression of ER-stress marker proteins, B) MKN74 mRNA expression of CRT, Gal-9 and PD-L1, C) total protein expression of CRT and PD-L1 in MKN7 and MKN74 cells. Bar graphs display the mean and SEM of at least 3 technical replicates.

Sup. Fig. 3: Chemoradiation treatment (full blot)

MKN7 MKN74

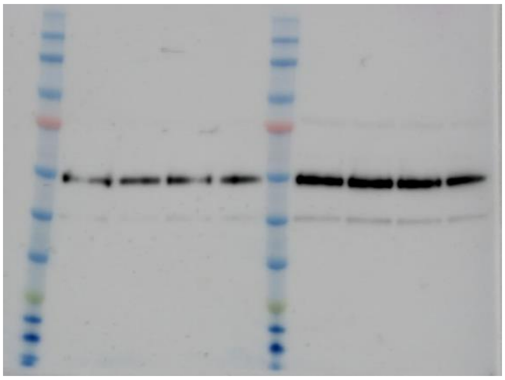

Calreticulin, ~55kDa

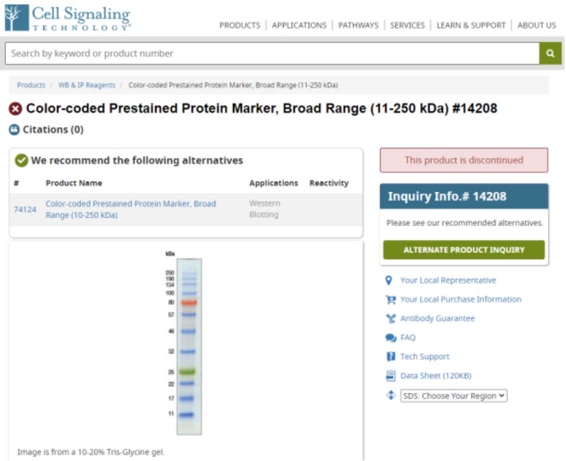

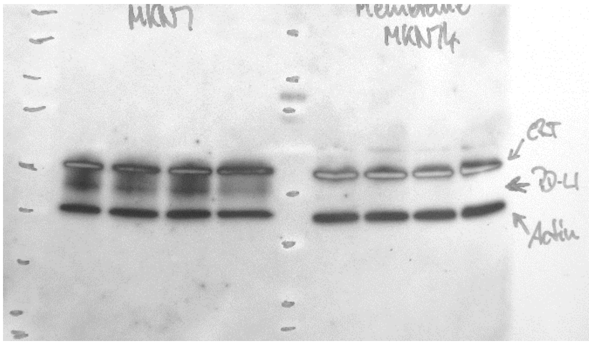

PD-L1

β-actin

untreated 4 hrs 24 hrs 48 hrs untreated 4 hrs 24 hrs 48 hrs

Sup. Fig.3: MKN7 and MKN74 were treated using 150uM or 300uM of 5FU, 20Gy of X-ray irradiation or chemoradiation and analysed 4, 24 and 48 hrs after single dose treatment. A) MKN74 mRNA expression of ER-stress marker proteins, B) MKN74 mRNA expression of CRT, Gal-9 and PD-L1, C) total protein expression of CRT and PD-L1 in MKN7 and MKN74 cells.

(Above) Calreticulin has been reblotted and analysed using anti-CRT Ab only to make sure there is no overlay with other signals.
